# Work of breathing, not dysoxia, as the cause of low central venous blood O_2_ saturation in sepsis

**DOI:** 10.1186/s13054-016-1476-1

**Published:** 2016-09-19

**Authors:** Guillermo Gutierrez

**Affiliations:** Pulmonary, Critical Care and Sleep Medicine Division, The George Washington University MFA, 2150 Pennsylvania Ave, NW, Washington, DC 20037 USA

The review by Nguyen et al. [1] acknowledges the substantially lower baseline central venous oxygen saturation (S_cv_O_2_) values reported by Rivers et al. [2] (48.6 ± 11.2 %) when compared to those for ProCESS [3] (71 ± 13 %), ARISE [4] (72.7 ± 10.5 %) and ProMISe [5] (64 ± 20 %) trials. Assuming normality, the distribution of baseline S_cv_O_2_ values in the study by Rivers et al. differed from those of the other trials (Fig. 1; *p* < 0.0001 by *t* test). Nguyen et al. ascribed this difference to “earlier central venous catheter (CVC) placement, greater shock severity or imbalances between O_2_ delivery and O_2_ consumption before corrective interventions”.

**Fig. 1:**
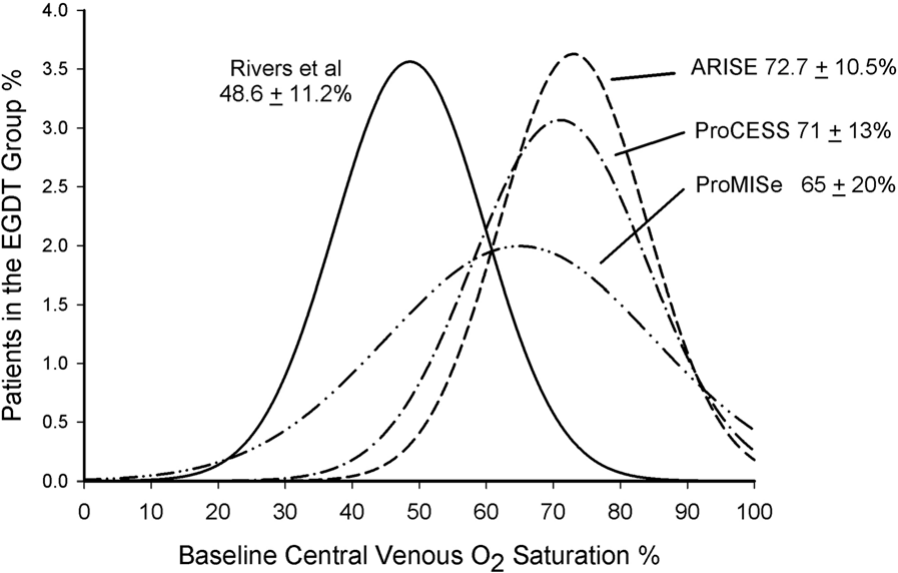
Gaussian distributions for baseline S_CV_O_2_ reported by Rivers et al [2], ProCESS [3], ARISE [4] and ProMISe trials [5]. Baseline S_CV_O_2_ was substantially lower in the Rivers et al trial when compared to each of the other trials (*p* < 0.0001)

One aspect of these trials that has been ignored up to now is the CVC position in the superior vena cava. According to accepted guidelines, the tip of the CVC should lie below the anterior first rib and above the right atrium, placing the tip of the CVC below the opening of the azygos vein, a vessel carrying venous blood from the intercostal muscles and portions of the diaphragm.

In the study by Rivers et al., 53.8 % of patients randomized to the early goal-directed therapy (EGDT) group required invasive mechanical ventilation during the first 6 h of treatment, a greater rate (*p* < 0.0001; Chi Square test) than those reported by ProCESS (26.4 %), ARISE (22.2 %), and ProMISe (19 %). Furthermore, the baseline respiratory rate for the EGDT cohort in Rivers et al. (31.8 ± 10.8 bpm) was greater (*p* < 0.001) than those reported by ProCESS (25.4 ± 7.0 bpm) and ARISE (24. ± 7.5 bpm). The baseline respiratory rate for patients in the ProMISe trial was not reported.

These data infer that patients in the study of Rivers et al. experienced considerable respiratory distress prior to the initiation of mechanical ventilation. This condition was likely associated with an increased work of breathing and the discharge of highly desaturated blood by the azygos vein into the superior vena cava, in close proximity to the fiber optic lumen of the catheter tip, precisely where S_cv_O_2_ was measured spectrophotometrically.

It is reasonable, therefore, to propose that the low S_cv_O_2_ values reported in the study of Rivers et al. reflected work by the muscles of respiration and not sepsis-associated systemic tissue dysoxia. In that instance, the S_cv_O_2_ increases observed during the first 6 h of treatment in the study by Rivers et al. may have been in response to unloading of respiratory muscles by mechanical ventilation and not to red blood cell transfusion or dobutamine infusion as proposed by their treatment algorithm.

## Abbreviations

CVC: Central venous catheter
EGDT: early goal-directed therapy
S_cv_O2: Central venous oxygen saturation
